# RECG Maintains Plastid and Mitochondrial Genome Stability by Suppressing Extensive Recombination between Short Dispersed Repeats

**DOI:** 10.1371/journal.pgen.1005080

**Published:** 2015-03-13

**Authors:** Masaki Odahara, Yuichi Masuda, Mayuko Sato, Mayumi Wakazaki, Chizuru Harada, Kiminori Toyooka, Yasuhiko Sekine

**Affiliations:** 1 Department of Life Science, College of Science, Rikkyo (St. Paul’s) University, Toshima-ku, Tokyo, Japan; 2 RIKEN Center for Sustainable Resource Science, Tsurumi, Yokohama, Kanagawa, Japan; Karlsruhe Institute of Technology, GERMANY

## Abstract

Maintenance of plastid and mitochondrial genome stability is crucial for photosynthesis and respiration, respectively. Recently, we have reported that RECA1 maintains mitochondrial genome stability by suppressing gross rearrangements induced by aberrant recombination between short dispersed repeats in the moss *Physcomitrella patens*. In this study, we studied a newly identified *P*. *patens* homolog of bacterial RecG helicase, RECG, some of which is localized in both plastid and mitochondrial nucleoids. *RECG* partially complements *recG* deficiency in *Escherichia coli* cells. A knockout (KO) mutation of *RECG* caused characteristic phenotypes including growth delay and developmental and mitochondrial defects, which are similar to those of the *RECA1* KO mutant. The *RECG* KO cells showed heterogeneity in these phenotypes. Analyses of *RECG* KO plants showed that mitochondrial genome was destabilized due to a recombination between 8–79 bp repeats and the pattern of the recombination partly differed from that observed in the *RECA1* KO mutants. The mitochondrial DNA (mtDNA) instability was greater in severe phenotypic *RECG* KO cells than that in mild phenotypic ones. This result suggests that mitochondrial genomic instability is responsible for the defective phenotypes of *RECG* KO plants. Some of the induced recombination caused efficient genomic rearrangements in *RECG* KO mitochondria. Such loci were sometimes associated with a decrease in the levels of normal mtDNA and significant decrease in the number of transcripts derived from the loci. In addition, the *RECG* KO mutation caused remarkable plastid abnormalities and induced recombination between short repeats (12–63 bp) in the plastid DNA. These results suggest that RECG plays a role in the maintenance of both plastid and mitochondrial genome stability by suppressing aberrant recombination between dispersed short repeats; this role is crucial for plastid and mitochondrial functions.

## Author Summary

Recombinational DNA repair plays an important role in the maintenance of genomic stability by repairing DNA double-strand breaks and stalled replication forks. However, recombination between nonallelic similar sequences such as dispersed repeated sequences results in genomic instability. Plant plastid and mitochondrial genomes are compact (generally approximately 100–500 kb in size), but they contain essential genes. A substantial number of repeats are dispersed in these genomes, particularly in the mitochondrial genome. In this study, we showed that a knockout mutation of the newly identified plant-specific homolog of bacterial RecG DNA helicase RECG caused some defects in plastids and significant defects in the mitochondria. The organelle genomes in these mutants were destabilized by induced aberrant recombination between short (<100 bp) dispersed repeats. Recombination was induced at repeats as short as 8 bp. This suggests that RECG maintains plastid and mitochondrial genome stability by suppressing aberrant recombination between short dispersed repeats. Because such a phenomenon, to our knowledge, has not been observed in bacterial *recG* mutants, our results suggest an organelle-specific genome maintenance system distinct from that of bacteria.

## Introduction

Plants have two organelles, plastid and mitochondrion, that possess their own genomic DNA. The organelle genomes have become compact due to the endosymbiotic transfer of ancestral bacterial genes into the nucleus throughout evolution [1]. However, their genomes still encode components essential for photosynthesis, respiration and gene expression in organelles [2]. Since electron transport in photosynthesis and respiration produce reactive oxygen species (ROS), a harmful factor that damages DNA, plant organelle DNA is exposed to more severe conditions than nuclear DNA. Ultraviolet (UV) radiation from sunlight can also damage organelle DNA. However, the mechanism of how plant organelle DNA stability is maintained remains largely unknown.

Nuclear genes involved in mtDNA stability have been identified through the analyses of mutants displaying variegated leaves or by mutating genes that were predicted to be involved in organelle DNA metabolism [3]. The bryophyte *P*. *patens* has two functional bacterial-type RecA homologs, RECA1 and RECA2, which localize to mitochondria and plastids, respectively [4,5]. A *RECA1* KO strain exhibits defects in growth and mitochondrial morphology, and results in lower rate of the recovery of damaged mtDNA [4,6]. Moreover, the *RECA1* KO mutant displays gross rearrangements due to aberrant recombination between short repeats ranging from 62 to 84 bp scattered throughout mtDNA, which suggests that RECA1 maintains mtDNA stability by suppressing gross rearrangements [6].

In the angiosperm *Arabidopsis thaliana*, a mutation in the MutS homolog 1 (*MSH1*) causes mtDNA instability due to aberrant recombination between short dispersed repeats ranging in size from 108 to 556 bp [7–10]. Similarly, mutations in plant-specific single strand DNA-binding proteins, *WHY2* from the whirly family of proteins [11] and organellar single-stranded DNA binding protein 1 (*OSB1*; [12]), lead to aberrant recombination between repeats. In the *OSB1* mutant, repeats ranging in size from 249 to 556 bp are involved in the recombination [12], while in the *WHY2* mutant, the recombination occurs between short repeats (<30 bp) and is gyrase inhibitor-dependent [11]. Mutations in *RECA3*, a RecA homolog, also cause mtDNA instability due to aberrant recombination between a few pairs of repeats (∼200 bp) [13].

A few genes are reported to be involved in the maintenance of plastid DNA (ptDNA) stability. Double mutations in *WHY1* and *WHY3*, whirly family genes in *A*. *thaliana*, induce recombination between 10–18 bp ptDNA repeats. Thus, WHY1 and WHY3 protect ptDNA against illegitimate recombination [14]. A recent report showed that a mutation in *A*. *thaliana MSH1* also induced rearrangements of plastid loci containing short repeats [15].

Bacterial RecG protein is a double-stranded DNA helicase that unwinds a variety of branched DNAs modeled after Holliday junctions and replication forks [16,17]. Analyses of a *recG* mutant suggest that RecG plays a role in homologous recombination and replication fork repair *in vivo*, similar to the proposed role of RecA [18]. In vitro studies also suggest the role of RecG in the repair of stalled replication forks [17,19,20] Recent reports suggest that RecG has an important function in the control of chromosomal replication and segregation in *E*. *coli* [21–23]. In this report, we analyzed a nuclear-encoded homolog of bacterial DNA helicase RecG, named RECG, which localized to both plastid and mitochondrial nucleoids in *P*. *patens*. We found that both organelle genomes of the *RECG* KO mutant were destabilized due to recombination between repeated sequences within a broad range in size (8–79 bp) and that the induced mtDNA recombination in *RECG* and *RECA1* KO mutants partly differed. Here, we propose a vital role for RECG in the maintenance of plastid and mitochondrial genome stability.

## Results

### *P*. *patens* RECG protein localizes to both plastid and mitochondrial nucleoids

**Fig 1:**
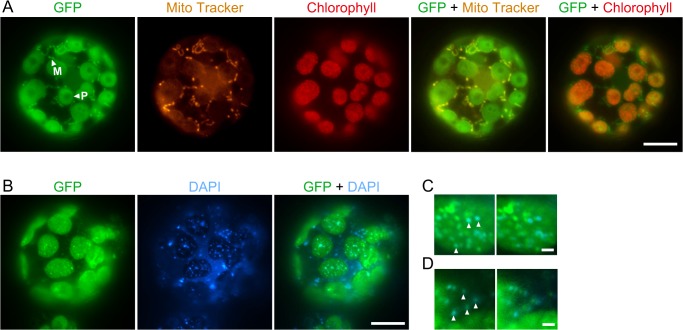
Subcellular localization of the RECG-GFP protein in *P*. *patens* protoplast cells. **A**. Subcellular localization of GFP fused to RECG N-terminal region. The fluorescence of GFP was merged with Mito Tracker or chlorophyll autofluorescence. Plastids were distinguished by the distribution of their chlorophyll autofluorescence, while mitochondria were detected by staining with Mito Tracker Orange. The arrowheads with P and M denote examples of RECG-GFP localized to plastid and mitochondrion, respectively. **B**. Subcellular localization of GFP fused to full-length RECG. The fluorescence of GFP was merged with DAPI fluorescence. **C** and **D**. Localization of full-length RECG-GFP to plastid (C) and mitochondrial (D) nucleoids. GFP fluorescence was merged with DAPI fluorescence (left panels) and then GFP fluorescence was shifted to left (right panels). The arrowheads denote examples of correspondence between GFP and DAPI signals. Bars = 10 μm in A and B, and 1 μm in C and D.

We identified a homolog of *E*. *coli* RecG in the *P*. *patens* nuclear genomic sequence [24] and named it *RECG*. Homologs of RecG are found in other plants, but not in fungi or animals, like bacterial-type RecA homologs [25]. Based on its cDNA sequence which we determined by rapid amplification of cDNA ends (RACE), the RECG protein is predicted to be 1152 amino acids in length and shares a high degree of sequence similarity with *E*. *coli* RecG, except for its extended N-terminal region (S1A Fig.). The extended N-terminal region, which is assumed to be a signal peptide that targets the protein to organelles, is potentially sufficient for localization to both plastid and mitochondrion (S1B Fig.) as judged by TargetP [26]. A similar N-terminal extension also exists in an annotated version of the RecG homolog in *A*. *thaliana* with a potential for localizing to both plastid and mitochondria (S1B Fig.). Fluorescent microscopy of protoplast cells expressing green fluorescent protein (*GFP*) gene fused to downstream of the 5’UTR and the N-terminus of *RECG* cDNA showed that the RECG-GFP localized to both plastids and mitochondria (Fig. 1A). Similar analysis with *GFP* gene fused to full-length *RECG* coding sequence demonstrated GFP fluorescence foci in both plastids and extra-plastid cytoplasmic space (Fig. 1B). 4′,6-diamidino-2-phenylindole (DAPI) staining of the cell showed that the GFP foci sometimes corresponded to some plastid and mitochondrial nucleoids (Fig. 1C and D), suggesting that RECG protein associates with these nucleoids.

### *RECG* partially complements the defects of *E*. *coli recG* cells

**Fig 2:**
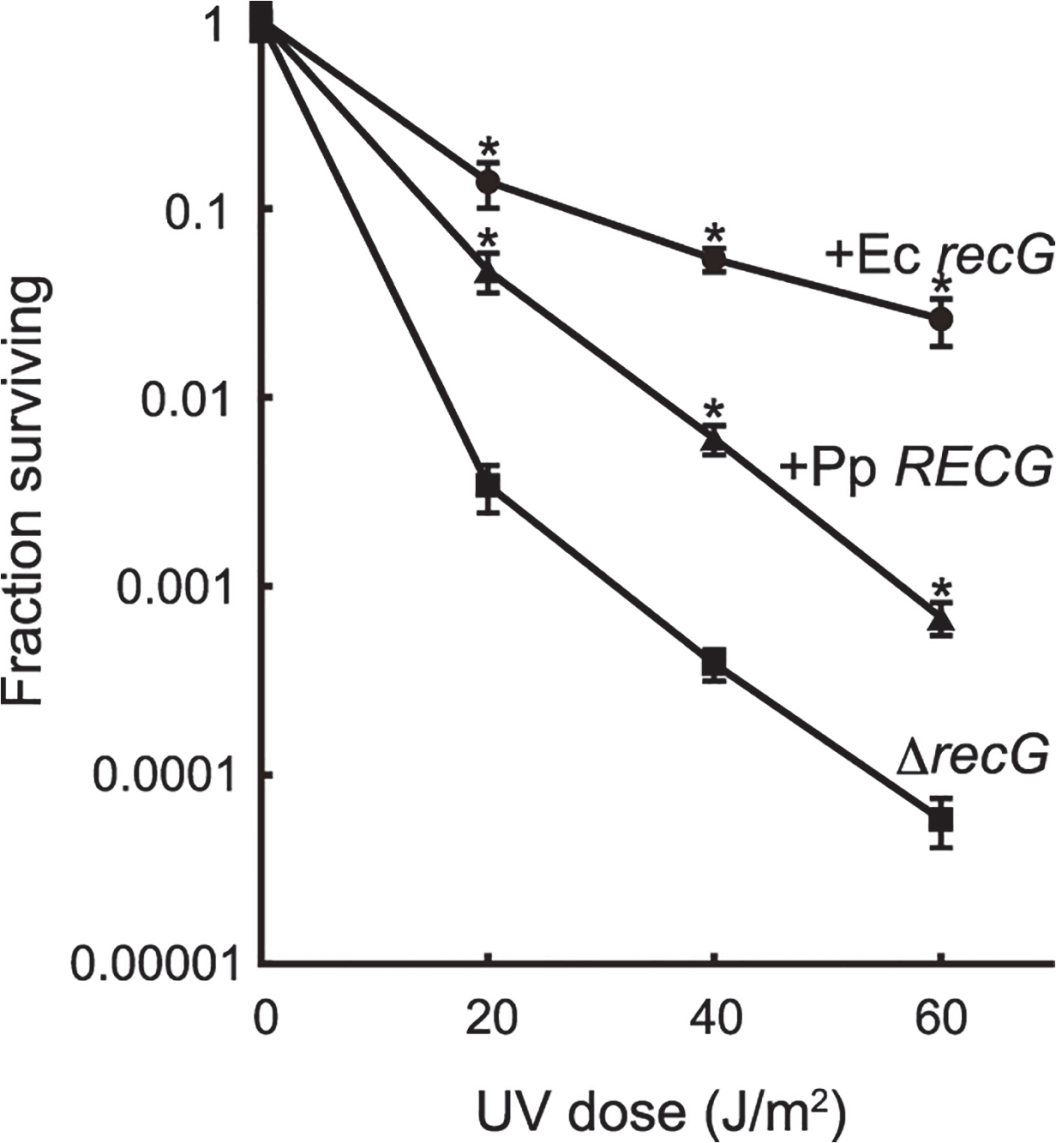
Complementation of the *E*. *coli recG* defect by *RECG*. *E*. *coli recG*-deficient cells harboring a plasmid carrying the *P*. *patens RECG* (+Pp *RECG*, triangle), *E*. *coli recG* (+Ec *recG*, circle) or empty vector (Δ*recG*, square) were subjected to UV irradiation, and the surviving fraction was calculated as described in Materials and Methods. Data from three independent experiments are expressed as mean ± SD. *p<0.01 (versus Δ*recG*).

To characterize the function of RECG, we examined whether *RECG* could complement the defects of an *E*. *coli recG*-deficient strain. *E*. *coli recG*-deficient strains harboring *P*. *patens RECG* lacking the signal peptide, intact *E*. *coli recG*, or no *recG* were subjected to UV irradiation after the induction of these genes. As reported by Ishioka *et al*. [27], the *recG*-deficient strain exhibited greater sensitivity to UV than the strain harboring the *recG* gene (Fig. 2), which implies that *E*. *coli* RecG participates in the recovery from UV damage. Expression of *P*. *patens RECG* conferred more than 10-fold greater resistance to UV in the *recG*-deficient cells, although not to the same degree as *E*. *coli recG* (Fig. 2). Therefore, *RECG* can partially complement the defects of *E*. *coli recG*-deficient cells.

### Knock-out of the *RECG* gene causes growth and developmental defects

**Fig 3:**
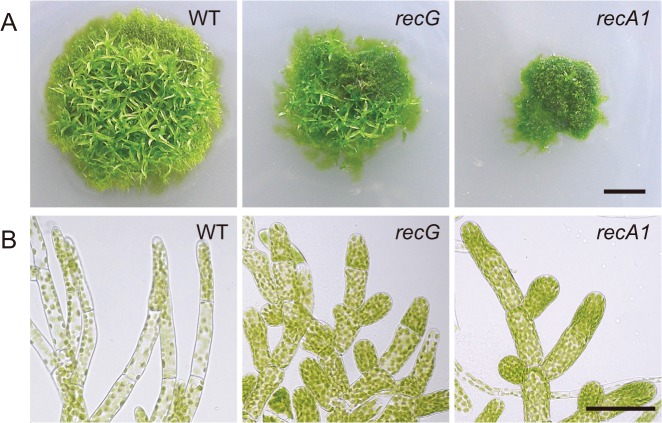
Cell growth and morphology of *RECG* KO plants. **A**. Colonies of wild type (WT), *RECG* KO (*recG*-2) or *RECA1* KO plants cultivated on agar medium for four weeks. **B**. Protonemal cells. Bars = 5 mm in (A) and 50 μm in (B). Atrophic protonemal cells (S3 Fig.) are shown as *RECG* KO cells.

Efficient targeting of nuclear genes [28] and a sequenced nuclear genome [24] enable easy knock-out of nuclear genes in *P*. *patens*. Thus, we knocked out the *RECG* gene to analyze the *in vivo* role of RECG (S2 Fig.). To investigate the effect of *RECG* KO on the growth and development of *P*. *patens*, we compared the *RECG* KO lines (named *recG*-1 and *recG*-2) with wild type (WT). After inoculation on agar medium, we observed that *P*. *patens* initially formed colonies composed of filamentous protonemal cells, and gametophores subsequently developed in the colonies. The *RECG* KO colonies appeared small and had less developed gametophores, which indicates defects in growth and development, although the extent of the defects were milder than those of the *RECA1* KO strain (Fig. 3A). The *RECG* KO colonies consisted of protonemal cells with heterogeneity in growth; relatively normal (*recG*-N) and atrophic (*recG*-A) protonemal cells. The *recG*-A protonemal cells were shorter and darker than the *recG*-N protonemal cells, while the *recG*-A cells are still shorter and darker than the WT (S3A Fig.). The atrophic protonemal cells of the *RECG* KO colonies were shorter than those of WT and were dense with plastids, likely due to a reduction in cell volume. Notably, the morphological abnormalities of the *RECG* KO and *RECA1* KO colonies were similar (Fig. 3B). These morphological effects imply that *RECG* plays an important role in the growth and development of *P*. *patens*, and suggest that *RECG* and *RECA1* share similar roles.

### Abnormal mitochondria and plastids in *RECG* KO cells

**Fig 4:**
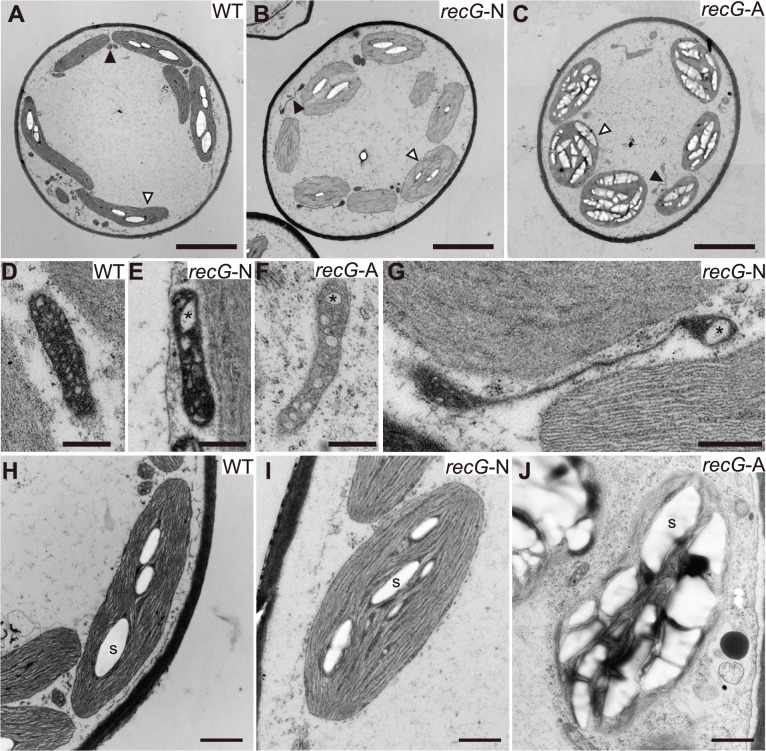
Ultrastructure of *RECG* KO protonemal cells. Protonemal cells of WT, *RECG* KO normal (*recG*-N), and *RECG* KO atrophic (*recG*-A) were analyzed by TEM. **A** to **C**. Images of transversion sections of WT (A), *recG*-6N (B), and *recG*-6A (C) cells. Filled and blank arrowheads denote examples of mitochondrion and plastid, respectively. **D** to **G**. Mitochondria of WT (D), *recG*-3N (E), *recG*-3A (F), and *recG*-6N (G) cells. Cristae were appeared as small regions with low electron density. Asterisks denote examples of enlarged cristae. **H** to **J**. Plastids of WT (H), *recG*-3N (I), and *recG*-3A (J) cells. Examples of starch grains are indicated by S. Bars = 5 μm in (A) to (C), 500 nm in (D) to (G), and 1 μm in (H) to (J).

To analyze the effect of the *RECG* KO on the ultrastructure of subcellular components, especially on those of mitochondria and plastids, we observed *RECG* KO cells by transmission electron microscopy (TEM). Since the *RECG* KO plant appeared to be composed of *recG*-N and *recG*-A protonemal cells, these two cell types were analyzed separately. TEM analyses revealed that the *RECG* KO had various effects on the ultrastructure of mitochondria, plastids, and other cell components. Both *recG*-N and *recG*-A mitochondria had a lower number of cristae and cristae enlargement (Fig. 4D-F), and *recG*-A cell mitochondria showed weaker matrix staining, indicating a lower electron density of the mitochondrial matrix (Fig. 4F). Some *RECG* KO mitochondria were abnormally extended (Fig. 4B, C and G), and their sizes were sometimes comparable to those of plastids (S3B Fig.). The extended mitochondria were more frequently observed in *recG*-N cells than in *recG*-A cells. It is notable that these mitochondrial abnormalities, including a lower number of cristae, cristae disorganization, weaker matrix staining, and stretching, are also observed in *RECA1* KO mitochondria [6]. We further analyzed the stretching by performing TEM on serial thin sections to elucidate the three-dimensional structure of the extended mitochondria, and found that one of these mitochondria penetrated 16 serial thin sections (S3C Fig.) and that the edge of each mitochondrion was swollen (S3D Fig.). This result suggested that the extended mitochondria were actually disc-shaped with thick edges.

The *RECG* KO affected plastids. Although plastids in *recG*-N cells looked mostly normal (Fig. 4I), abnormally extended plastids were occasionally detected. Some of these plastids appeared to have a disturbance in cell division septum formation (S3E Fig.). Plastids in *recG*-A cell had abnormal shapes, and their membranes, including thylakoid, outer and inner membranes, appeared frail (Fig. 4J). Moreover, most plastids in *recG*-A cells abundantly accumulated starch and had an underdeveloped thylakoid membrane (Fig. 4C and J). The *RECG* KO also affected cell structure and cell components, especially in *recG*-A cells. Some *recG*-A protonemal cells accumulated oil (S3F Fig.), which is in contrast to wild type protonemal cells, which have few or no oil bodies [29]. These observations indicate that *RECG* KO had various effects on cell ultrastructure that were likely caused by functional defects in mitochondria and plastids.

### Mitochondrial DNA rearrangements in *RECG* KO plants

In *RECA1* KO mitochondria, aberrant recombination occurs frequently between repeats ranging in size from 62 to 84 bp and results in gross DNA rearrangements, which appear to be responsible for the phenotypic defects observed in *RECA1* KO plants [6]. Therefore, the similar phenotype of *RECG* and *RECA1* KO plants, as described above, predicts that *RECG* KO mtDNA also undergoes gross DNA rearrangements. To test this hypothesis, we carried out structural analyses of *RECG* KO mtDNA and compared the results with those of *RECA1* KO mtDNA.

**Fig 5:**
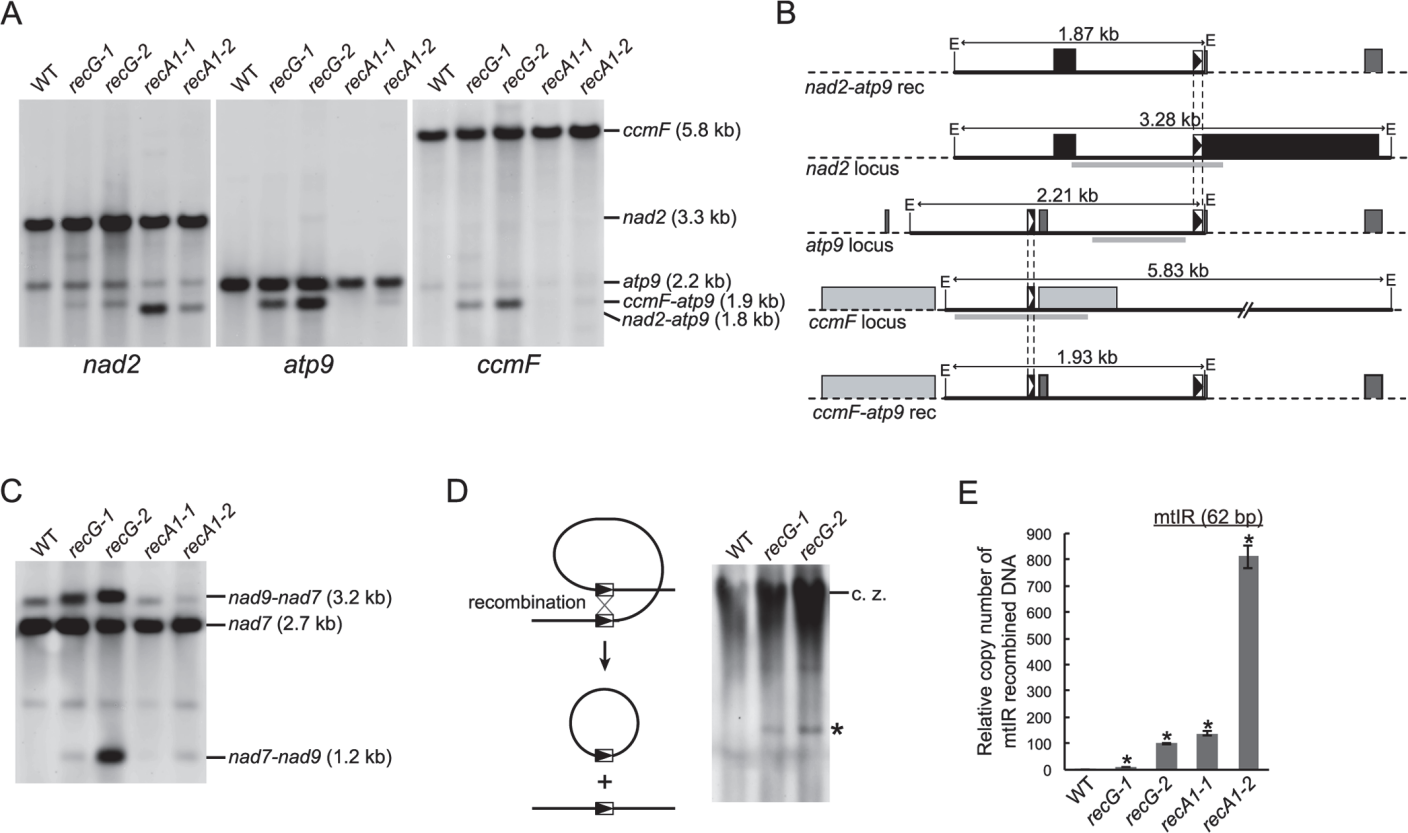
mtDNA rearrangements in *RECG* KO lines. **A**. mtDNA configuration at *nad2*, *atp9* and *ccmF* loci. DNA from WT, *RECG* KO and *RECA1* KO strains digested with *Eco*RI was probed using *nad2*, *atp9* and *ccmF* probes, as indicated below the blots. The predicted structure and length of the major bands are indicated on the right. **B**. Schematic representation of the DNA structures detected in (A). The EcoRI fragments corresponding to the bands on the blot in (A) and the flanking regions are represented by solid black lines and dashed black lines, respectively. The EcoRI recognition sites are indicated by E. The positions of the probes used in (A) are indicated by thick gray lines. The boxes represent exons, and the lines between boxes represent introns or noncoding flanking sequences. The 69 bp and 47 bp repeats are indicated by black triangles and white triangles in the boxes, respectively. **C**. mtDNA configuration at the *nad7* locus containing 79 bp repeats. DNA from each of the indicated strains was digested with SacII and probed using *nad7* probe. The structure of the fragments is detailed in Odahara et al. [6]. **D**. Production of deleted mitochondrial subgenome by recombination between repeats in *nad7* and *nad9*. Left panel illustrates production of deleted subgenome by intramolecular recombination between direct repeats. Undigested DNA from WT and *RECG* KO strains was probed using *nad7* probe. The asterisk denotes DNA corresponding to 11-kb subgenome. c.z., compression zone. **E**. The amount of DNA generated by recombination between mtIR (62 bp). Relative copy number of DNA resulting from recombination between mtIR per mitochondrial *rpl2* DNA was measured by qPCR. WT was given a value of 1. The data represent mean of three replicates ± SD. *p<0.01 (versus WT).

A product resulting from recombination between 69 bp direct repeats existing in *nad2* and *atp9* loci of mtDNA, which appears as a 1.8 kb *Eco*RI fragment on DNA gel blots [6], was confirmed in a blot hybridized with *nad2* probe in *RECA1* KO lines (Fig. 5A). The blot showed that the 1.8 kb *Eco*RI fragment of the *nad2*-*atp9* recombination product was hardly detectable in the *RECG* KO lines, while a weak 1.9 kb band was detected in both *RECG* KO lines (Fig. 5A). Since the *RECG* KO-specific 1.9 kb DNA fragment strongly hybridized to both an *atp9* probe, which does not hybridize to the *nad2*-*atp9* recombination product, and a *ccmF* probe (Fig. 5A), this fragment is likely to be the result of recombination between the *atp9* locus and *ccmF* locus. Forty-seven base pair repeats at both loci are suspected to be involved in recombination (Fig. 5B and S4D Fig.), and the size of the recombination product is consistent with the size of the observed 1.9 kb *ccmF*-*atp9* product. Because the *ccmF*-*atp9* recombination product includes the 69 bp *nad2-atp9* repeats (Fig. 5B), it hybridized weakly to the *nad2* probe (Fig. 5A). The *ccmF*-*atp9* product also appeared in the *RECG* KO lines with the sizes of the DNA products resulting from recombination between the 47 bp repeats when the mtDNAs were digested with HindIII or NdeI (S4A–C Fig.). PCR amplification of the *ccmF*-*atp9* product from *RECG* KO plants and direct sequencing analysis of the amplified fragments showed that almost all of the recombination junctions were within the 47 bp repeats, yet the sequence similarity extended to the region flanking the repeat (S4D Fig.). These results indicate that the *ccmF*-*atp9* recombination product, but not the *nad2-atp9* recombination product, accumulates in the *RECG* KO lines.

Next, we analyzed whether other hotspots identified in *RECA1* KO mtDNA rearrangements [6] also induced recombination in the *RECG* KO plants. DNA gel blot analysis showed that recombination occurred between the 79 bp *nad7*-*nad9* direct repeats in the *RECG* KO lines as well as the *RECA1* KO lines (Fig. 5C). In the *RECG* KO lines, we also identified signals corresponding to 11 kb of deleted circular mtDNA (Fig. 5D), which is produced by recombination between the *nad7*-*nad9* repeats, as reported in *RECA1* KO plants [6]. In addition, we carried out quantitative PCR (qPCR) analyses to assess the copy number of DNA resulting from recombination between 62 bp inverted repeats (mtIR), another hot spot located in the intergenic region of mtDNA [6]. The results showed that the recombination at this locus was induced in the *RECG* KO lines and that the recombination level of one line was comparable to that of the *RECA1* KO line (Fig. 5E). These results suggest that frequent mtDNA rearrangements, which associated with deletion in some cases, occur at multiple hot spots in *RECG* KO plants, and that some hot spots differ from those of the *RECA1* KO plants.

Since the recombination described above can cause deletion of mtDNA, it is possible that the copy number of *RECG* and *RECA1* KO mtDNA loci altered. To test this, we measured the copy number of three mitochondrial loci, *rps4*, *nad6* and *rpl2*, by qPCR. The results showed that the copy number of each mtDNA locus varied in the *RECA1* KO lines whereas increased in the *RECG* KO lines (S4E Fig.).

### Repeat-mediated genomic instability in *RECG* and *RECA1* KO mitochondria

**Fig 6:**
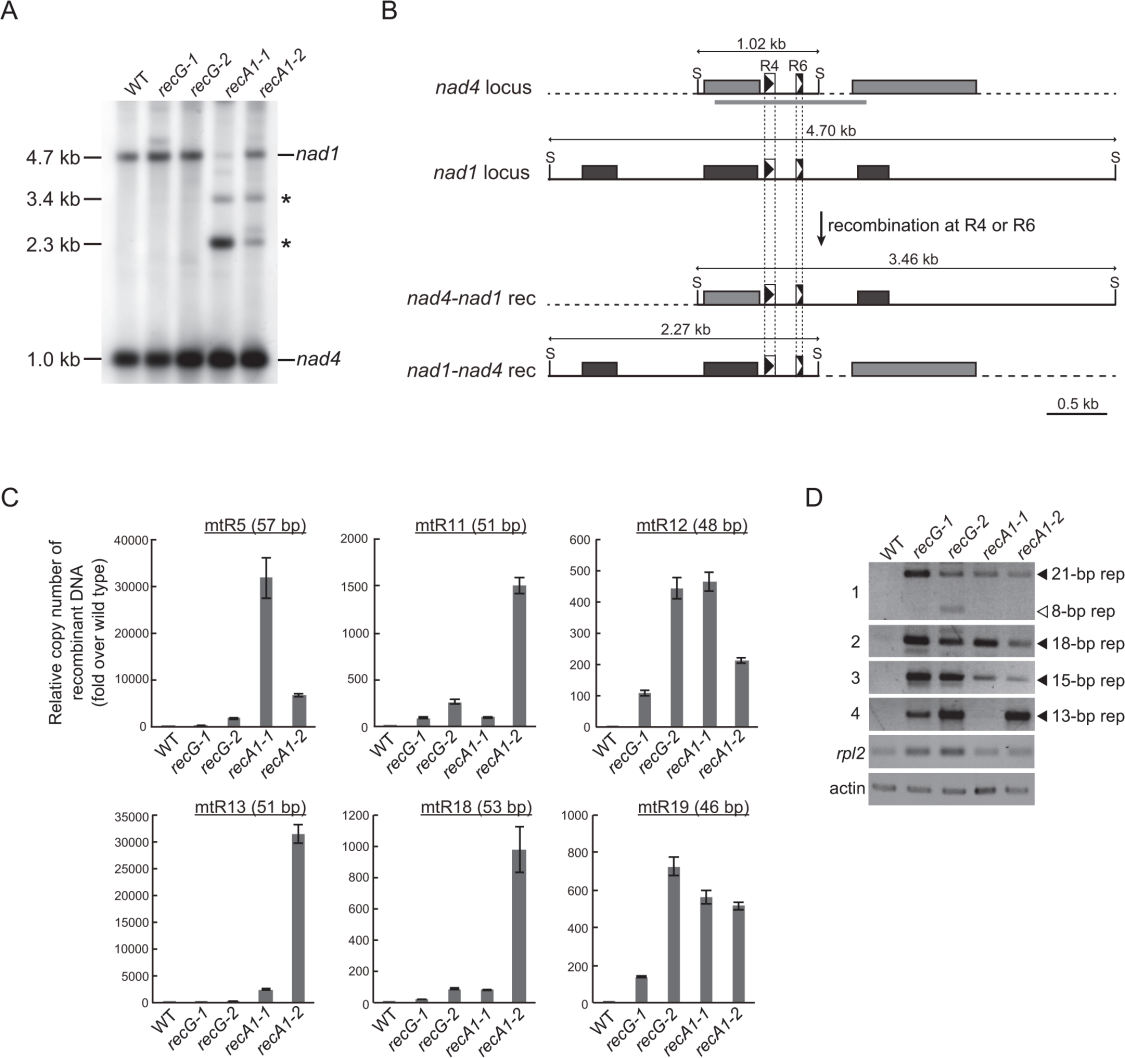
Genomic instability in *RECG* and *RECA1* KO mitochondria. **A**. mtDNA configuration at the *nad4* locus. DNA from WT, *RECG* KO, and *RECA1* KO strains digested with SacII were probed with *nad4* probe. The asterisks denote signals corresponding to DNA recombined between *nad4*-*nad1* repeats. The length of the bands is indicated on the left. **B**. Schematic explanation of the DNA structures detected in (A). Reciprocal recombination at R4 (90 bp) or R6 (56 bp) at *nad4* and *nad1* loci produces both *nad4*-*nad1* rec and *nad1*-*nad4* rec products. The SacII recognition sites are indicated by S. The positions of the probes used in (A) are indicated by thick gray lines. For details of the scheme, see legend of Fig. 5B. **C**. The amount of DNA generated by recombination between several direct repeats 46 to 57 bp in length. Relative copy number of DNA resulting from recombination between direct repeats (R5, R11, R12, R13, R18, or R19) per mitochondrial *rpl2* DNA was measured by qPCR. WT was given a value of 1. The data represent mean of three replicates ± SD. All the *RECG* KO and *RECA1* KO values are significantly different from WT values (p<0.01). **D**. DNA generated by recombination between short (<35 bp) repeats. PCR reaction numbers indicated on the left correspond to those in S1 Table. Mitochondrial gene *rpl2* and nuclear gene actin were amplified as a control. Filled and blank triangles indicate DNA with the expected and unexpected sizes, respectively.

To analyze the effect of *RECG* or *RECA1* KO on global structure of mtDNA, we performed a comprehensive analysis of DNA molecules resulting from recombination between repeats dispersed in the mtDNA. We first analyzed mtDNA repeat-mediated rearrangements in both KO mutants by DNA gel blot, and identified two DNA fragments that were most likely to be derived from recombination between *nad4*-*nad1* direct repeats, named R4 (90 bp) or R6 (56 bp) [6], as judged by their sizes (Fig. 6A). Note that the DNA fragments were detected only in *RECA1* KO lines, but not in *RECG* KO line as well as WT. We next carried out quantification of DNA resulting from recombination between other direct repeats (46–57 bp, R5, R11, R12, R13, R18, and R19) by qPCR. The results showed that recombined DNA from every tested repeats were apparently accumulated in both *RECG* and *RECA1* KO lines; the level of accumulation was very high in the *RECA1* KO lines regarding R5 and R13, and high in *RECA1* KO lines regarding R11 and R18 (Fig. 6C). Collectively, these results suggest that the repeat-mediated recombination were induced in both *RECG* and *RECA1* KO mitochondria at multiple loci, but the degree and the site of the recombination were somewhat different between them.

To understand further the effect of *RECG* and *RECA1* KO on mtDNA stability, we examined whether shorter repeats were involved in the mtDNA instability. REPuter, a program that detects repeated sequences in DNA sequence [30], identified approximately 900 pairs of repeats (15–35 bp) in *P*. *patens* mtDNA [4]. Among the repeats, we analyzed selected repeats (S1 Table) by PCR with respect to the accumulation of recombination products, and the levels of DNA amplification at each repeat are shown in Fig. 6D. A greater level of amplification was observed in both KO lines than in the WT strain. Sequencing of the amplified DNA confirmed that they were the products of recombination between the 21, 18, 15 or 13 bp inverted or direct repeats (S1 Table and S8 Fig.). Moreover, the analysis further showed that an additional region of DNA, which was the product resulting from recombination between 8 bp repeats that are distantly positioned around the 21 bp repeats, was amplified in the *RECG* KO lines (Fig. 6D, panel 1). Therefore, DNA accumulates as a result of recombination between repeats ranging from 8 to 21 bp in the *RECG* KO, and from 13 to 21 bp in the *RECA1* KO mitochondria.

### Decrease in the levels of specific mitochondrial gene transcripts in *RECG* KO plants

**Fig 7:**
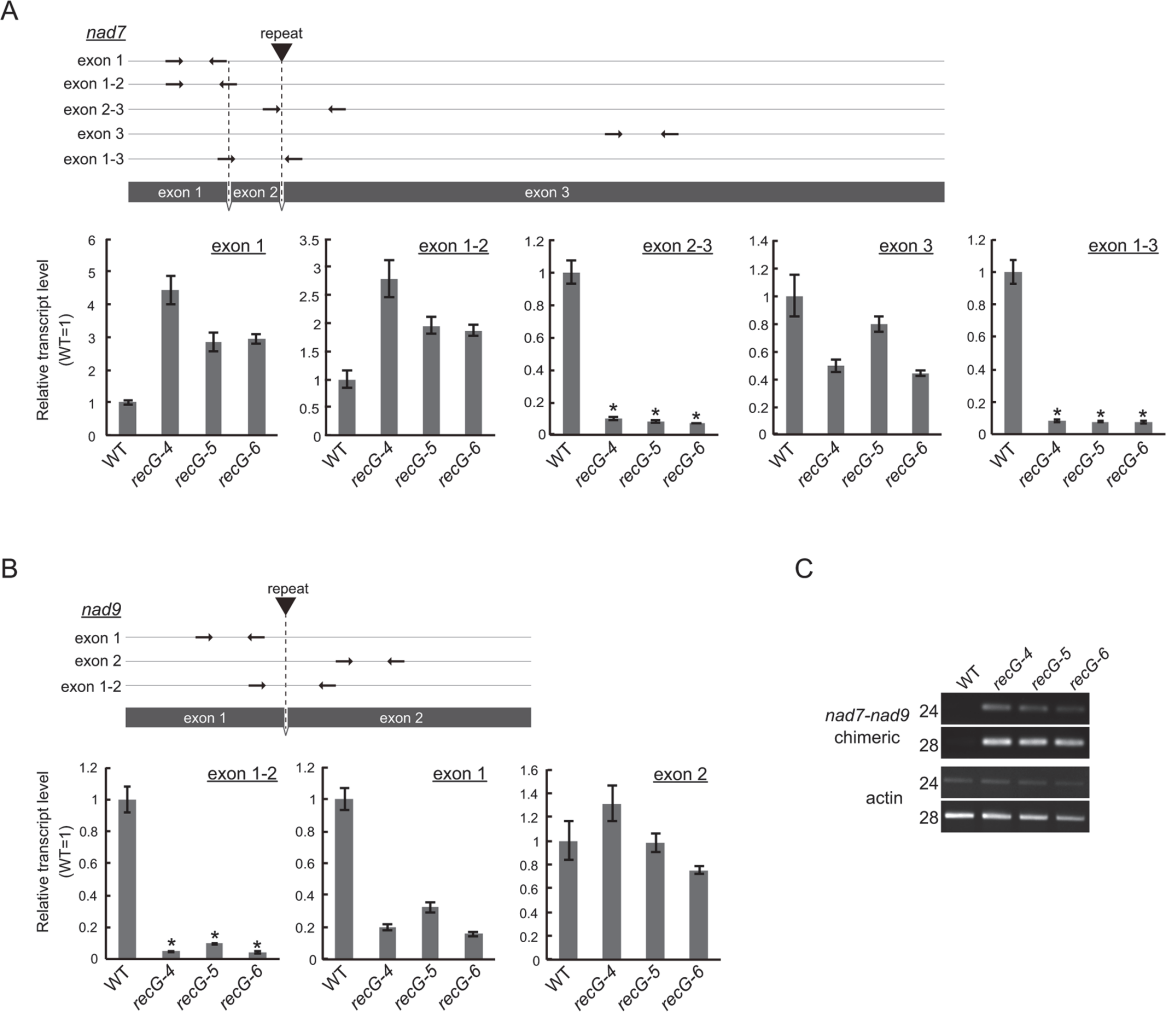
Mitochondrial transcripts in *RECG* KO plants. **A** and **B**. Detailed qRT-PCR analysis of *nad7* (A) and *nad9* (B) transcripts. Positions of the primers used in the qPCR are schematically represented in the upper parts of the panels. Coding regions are represented by grey boxes. Positions and directions of the primers are shown by arrows. Positions of the repeats involved in the rearrangements between *nad7* and *nad9* are indicated by triangles. Relative levels of segments of mitochondrial transcripts from *nad7* and *nad9* were normalized to reference of nuclear gene ST-P 2a transcript. WT was given a value of 1. Slight or no amplification was observed in no reverse-transcription controls (S5G Fig.). The data represent mean of three replicates ± SD. *p<0.01 (versus WT). **C**. RT-PCR analysis of *nad7-nad9* chimeric transcripts. *nad7-nad9* chimeric transcripts were amplified using cDNA from WT and *RECG* KO lines at cycles indicated on the left of the picture. Actin was amplified as an internal control.

The data presented here showed efficient rearrangements of *RECG* KO mtDNA at *nad7*, *nad9*, *atp9*, and *ccmF* loci caused by recombination between short repeats. Quantitative analysis using DNA gel blots revealed that the copy number of normal mtDNA bands (e.g., 1.5 kb of *nad7* band and 1.7 kb *nad9* band in S5A and B Fig.) in the *RECG* KO plants decreased to approximately 30%–45% and 35%–50% of WT at *nad7* and *nad9* loci, respectively. The copy number of normal mtDNA bands at *atp9* and *ccmF* loci did not significantly change (S5C and D Fig.). To investigate the effect of the mtDNA rearrangements on mitochondrial transcripts, we analyzed the transcripts of these loci in the *RECG* KO plants. Quantitative RT-PCR (qRT-PCR) analysis demonstrated a significant reduction in the levels of transcripts from *nad7* and *nad9* for some of the primer pairs in the *RECG* KO mutants (Fig. 7A and B). We found a significant reduction (<10% of WT levels) in the levels of the transcript fragments when the primers were arranged to amplify a segment including a junction of exon 2 and 3 for *nad7* or exon 1 and 2 for *nad9* (Fig. 7A and B). Because the introns contain repeats involved in the mtDNA rearrangements (Fig. 7A and B), these results suggest that a substantial number of the *nad7* and *nad9* transcripts exist as chimeric transcripts of *nad7* and *nad9* and not as individual intact forms. RT-PCR analysis demonstrated the efficient amplification of *nad7-nad*9 chimeric transcripts from *RECG* KO mutants (Fig. 7C). We confirmed that the chimeric transcripts were precisely spliced between *nad7* exon2 and *nad9* exon2 (S5E Fig.). In contrast, similar qRT-PCR analysis demonstrated no significant differences in the levels of transcripts from *ccmF* and *atp9* loci between the WT and *RECG* KO mutants (S5F Fig.). These results suggest that the efficient rearrangements of some mtDNA loci were associated with a decrease in the normal levels of mtDNA and a significant decrease in the number of the corresponding intact transcripts.

### Repeat-mediated genomic instability in *RECG* KO plastids

**Fig 8:**
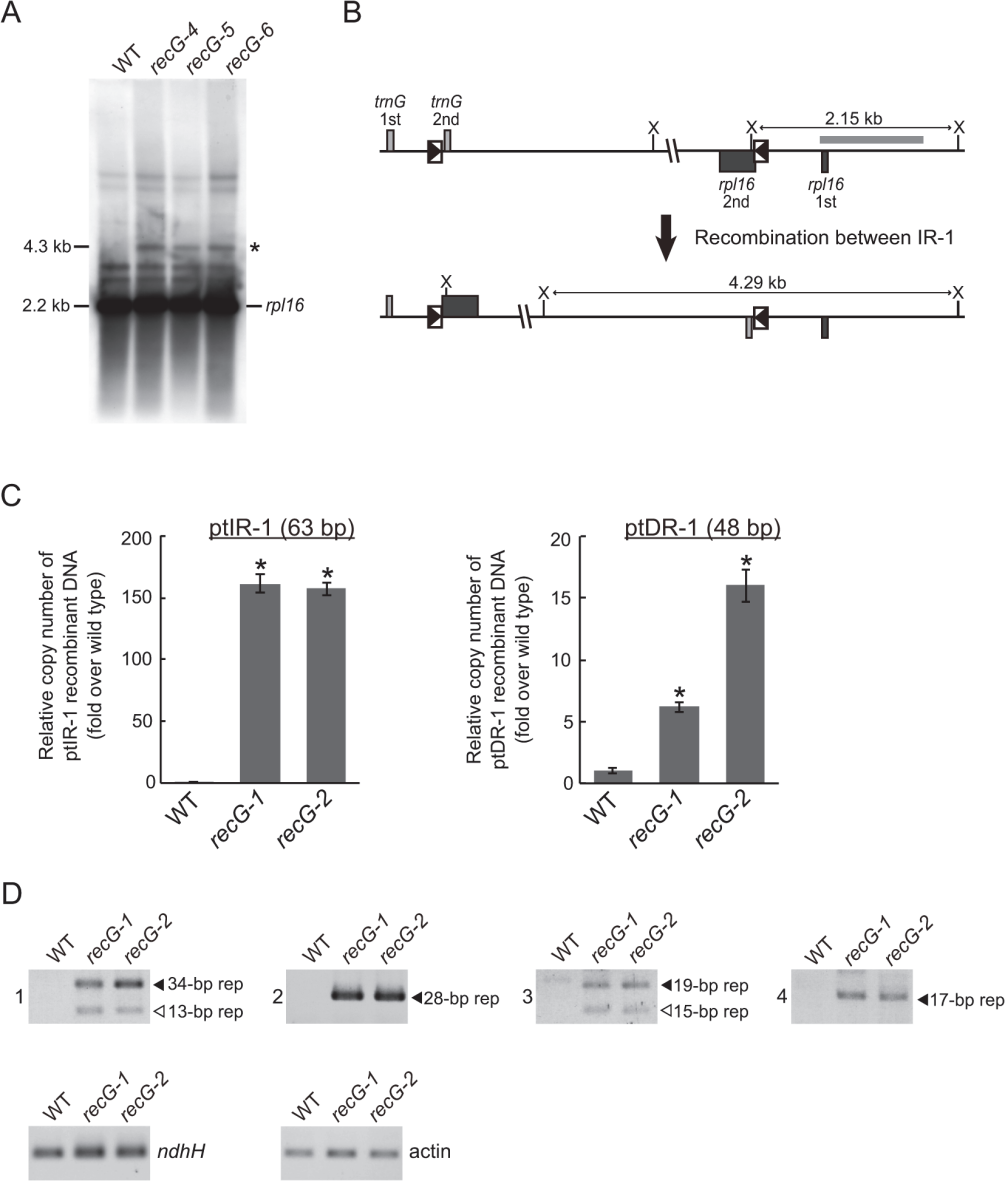
Genomic instability in *RECG* KO plastids. **A**. ptDNA configuration at the ptIR-1 (*rpl16*) locus. DNA from WT and *RECG* KO strains digested with XhoI were probed with *rpl16* probe. The asterisk denotes signals corresponding to DNA recombined between ptIR-1 repeats. The length of the bands is indicated on the left. **B**. Schematic explanation of the DNA structures detected in (A). Intramolecular recombination between ptIR-1 causes inversion of a segment between ptIR-1. The XhoI recognition sites are indicated by X. The boxes represent exons, and the lines between boxes represent introns or noncoding flanking sequences. ptIR-1 are indicated by black triangles in the boxes. The positions of the probes used in (A) are indicated by thick gray line. **C**. The amount of DNA generated by recombination between ptIR-1 (63 bp) or ptDR-1 (48 bp). Relative copy number of DNA per plastid *ndhH* DNA was measured by qPCR. WT was given a value of 1. The data represent mean of three replicates ± SD. *p<0.01 (versus WT). **D**. DNA generated by recombination between short (<35 bp) repeats. PCR reaction numbers indicated on the left of the pictures correspond to those in S3 Table. Plastid gene *ndhH* and nuclear gene actin were amplified as a control. Filled and blank triangles indicate DNA with the expected and unexpected sizes, respectively.

The fact that RECG protein not only localizes to mitochondria, but also to plastids, raises the possibility that RECG plays a role in both plastids and mitochondria. We then analyzed the structure of *RECG* KO ptDNA, and focused on recombination between repeated sequences. We searched for repeats using REPuter and identified 16 pairs of repeats longer than 40 bp in the *P*. *patens* ptDNA sequence [31], most of which were located immediately downstream of genes as palindromic sequences (S2 Table), probably functioning in the stabilization of transcripts [32]. Among the repeats, we analyzed the level of recombination between inverted repeats-1 (ptIR-1, 63 bp long, shown as R6 in S2 Table), which are located in *rpl16* and *trnG*, or direct repeats-1 (ptDR-1, 48 bp long, shown as R12 in S2 Table), which are located in *psaA* and *psaB* (S6A Fig.). DNA gel blot analysis using a plastid *rpl16* probe showed accumulation of 4.3 kb DNA fragments in the *RECG* KO mutants (Fig. 8A). The size of these DNA fragments corresponds to that of a predicted product resulting from recombination between ptIR-1 (Fig. 8B). We next analyzed the IR-1 recombination product using qPCR (S6 Fig.). The analyses revealed that the product formed by recombination between ptIR-1 showed ∼160-fold increase in the *RECG* KO mutants (Fig. 8C). Similar qPCR analysis of a product formed by recombination between ptDR-1 showed a 6–16-fold increase in the *RECG* KO mutants compared with WT (Fig. 8C). These results showed increased accumulation of ptIR-1 and ptDR-1 recombination products in the *RECG* KO lines. To assess the effect of *RECG* KO on copy number of ptDNA, qPCR analysis of ptDNA loci was performed. The copy number of three plastidic loci *rbcL*, *atpA* and *ndhH* showed increases in the *RECG* KO lines compared with WT (S6B Fig.). As the *RECG* KO mutants showed increase of ptDNA in every tested locus, it is possible that plastid number was increased in the *RECG* KO mutants. However, no significant difference was observed in the number of plastids per cell between WT and *RECG* KO mutants (S6C Fig.).

In the analyses described above, we applied PCR amplification for detection of DNA recombined between repeated sequences in the *RECG* or *RECA1* KO lines. However, since such a recombined DNA can be created during PCR reaction, named PCR jumping, as reported by Alverson et al. [33], we quantified the amount of the artificially recombined DNA in our qPCR assay to evaluate the effect of PCR jumping. We first prepared two DNA fragments that contain copy1 or copy2 of ptIR-1, and then the two fragments were mixed so as to contain the amount of each IR-1 copy equivalent to that of the WT total genomic DNA (S6D Fig.). Next we quantified copy number of DNA recombined between the copy1 and copy2 of ptIR-1 using WT genomic DNA or the mixed DNA as templates. The amount of DNA recombined between IR-1 from the mixed DNA by PCR jumping was ∼1.5% of that from WT genomic DNA (S6D Fig.), indicating that recombination between IR-1 copies occurred during the qPCR reaction, but the efficiency was very low. Accordingly, these results suggest that the amplified recombined DNA that we observed was mostly derived from in vivo recombination of organelle DNA, and that the contribution of in vitro recombination, if it occurred, was very small.

We extended the analyses to shorter repeats (15–35 bp), which are abundant (approximately 2000 pairs) in ptDNA. We carried out PCR analyses to estimate the amount of DNA that recombined between short repeats. Reliable DNA amplification occurred only in the *RECG* KO lines (Fig. 8D), and sequencing of the amplified DNAs demonstrated that they were the result of recombination between the 34, 28, 19 or 17 bp repeats (S3 Table and S8 Fig.). Moreover, some types of additional DNA, which were determined to be products of recombination between 13 bp or 15 bp repeats (S3 Table), were amplified only in the *RECG* KO lines (Fig. 8D). Collectively, these results suggest that genomic instability was induced in the *RECG* KO plastids by aberrant recombination among repeated sequences, ranging in size from 13 to 63 bp.

### Increased accumulation of recombined mtDNA in *recG*-A cells

**Fig 9:**
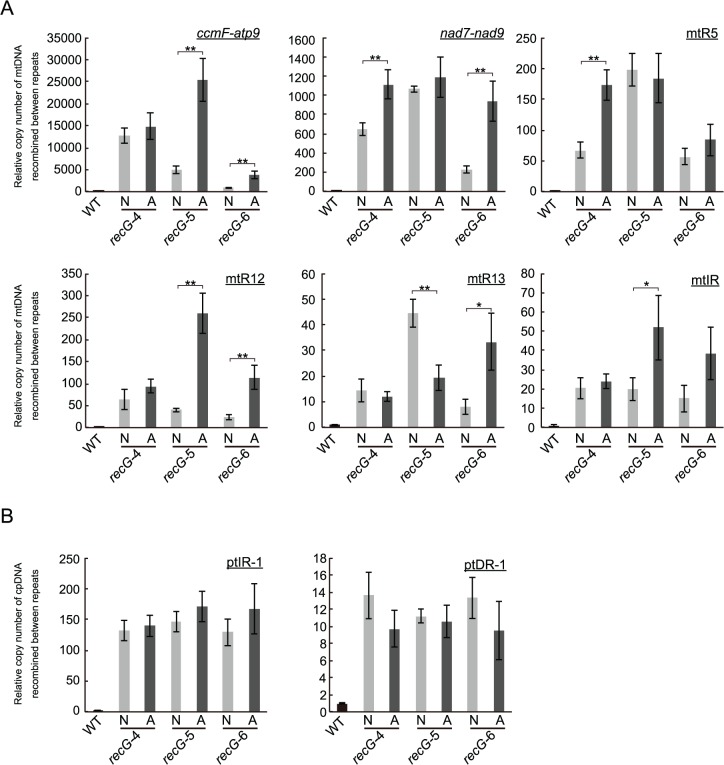
Status of organelle DNA in *recG*-N and *recG*-A cells. Relative copy number of DNA resulting from recombination between mitochondrial short repeats (*ccmF-atp9*, *nad7-nad9*, mtR5, mtR12, mtR13, and mtIR; A) per mitochondrial *rpl2* and plastidic short repeats (ptIR-1 and ptDR-1; B) per plastid *ndhH* in cells mainly comoposed of *recG*-N or *recG*-A cells were measured by qPCR using three independent *RECG* KO lines. WT was given a value of 1. The data represent mean of three replicates ± SD. *p<0.05, **p<0.01.

To investigate the relationship between the heterogeneity of atrophic phenotype of *RECG* KO plants appearing as *recG*-A and *recG*-N cells (S3A Fig.) and the stability of plastid and mitochondrial genomes, we compared the status of organelle DNA in *recG*-A and *recG*-N cells. We separately extracted total genomic DNA from protonemal cells mainly composed of *recG*-A cells or *recG*-N cells and measured the amount of mtDNA and ptDNA resulting from recombination between short repeats, using qPCR as described above (Fig. 6C and 8C). qPCR analyses of mtDNA showed that the number of the DNA molecules resulting from recombination between most of the tested repeats was higher in *recG*-A cells than that in *recG*-N cells. The levels of these recombination products in *recG*-N cells were still higher than those in WT cells (Fig. 9A). The levels of recombination product from *ccmF*-*atp9* repeats (originally identified as repeats involved in the mtDNA instability, Fig. 5A) and R12 significantly increased in *recG*-A cells (Fig. 9A). However, the qPCR analysis of ptDNA showed no significant difference between the amounts of ptIR-1 or ptDR-1 recombination products in *recG*-A and *recG*-N cells (Fig. 9B).

## Discussion

In this report, we showed that knocking out a plant-specific RecG homolog RECG induced genomic instability due to repeat-mediated recombination in both mitochondrial genome and plastid genome of *P*. *patens*. The induction of organelle genome recombination by *RECG* KO implies that organelle genomes can potentially undergo repeat-mediated recombination under normal culture conditions. Indeed, plant mitochondrial genomes are occasionally rearranged by recombination between short (<1 kb, in most cases <200 bp) repeats [34], and double-strand breaks in the plastid genome are repaired efficiently by utilizing recombination between short (<100 bp) repeats [35,36], both of which could contribute to potential genome rearrangements in plant organelles. Repeat-mediated recombination similar to those described in this paper were not observed in the bacterial *recG* mutants as far as we know. We propose that an important role of RECG is to maintain organelle genome stability by suppressing recombination among dispersed repeats.

Analysis of RECG-GFP subcellular localization revealed that the product(s) from the *RECG* gene localized to both plastids and mitochondria (Fig. 1). Examples of product(s) from a single gene being targeted to both plastid and mitochondrion, named dual targeting, have been reported and classified into two types [37]: an ambiguous signal peptide that can be recognized by both plastid and mitochondrion, or multiple N-terminal signal peptides for different organelles that are produced by alternative translation initiation and/or alternative splicing. Either type may account for the dual targeting of RECG, since *RECG* has two in-frame AUG codons in its 5’part that could localize their products to both organelles (S1B Fig.). Since *A*. *thaliana* RecG homolog also has the potential to localize to both organelles, as determined by our in silico analysis, dual targeting of RecG homolog may be conserved in both *P*. *patens* and *A*. *thaliana*.

Full-length RECG-GFP formed foci in plastids and mitochondria, and the foci sometimes corresponded to organelle nucleoids. This suggests that RECG is not a constitutive nucleoid protein but associates with organelle nucleoids with a bias. The nucleoid-associating RECG-GFP may be the functional RECG interacting with organelle DNA to maintain genomic stability.

Intriguingly, the *RECG* KO plants exhibited similar defects in growth, cell morphology, and mitochondrial morphology/activity to those of *RECA1* KO plants (Figs. 3 and 4). Mitochondrial defects including disorganized cristae, a lower electron density of the matrix, and an enlarged disc-shape, were also characteristic phenotypes of *RECA1* KO plants. The lower electron density of the matrix suggests lower mitochondrial activity, and was frequently observed in *recG-*A cells, suggesting that mitochondrial dysfunction was the cause of the atrophic phenotype. On the other hand, disorganized cristae and the enlarged disc-shaped mitochondria were commonly observed in *recG*-A and *recG*-N cells. As some of the disc-shaped mitochondria exhibited a normal matrix electron density (Fig. 4 and S3 Fig.), mitochondrial dysfunction was probably not responsible for the disc-shape. Disc-shaped mitochondria have been reported to occur in tobacco cells in response to low oxygen pressure without loss of membrane potential [38]. Thus, the disc-shaped mitochondria we observed may reflect the dynamics of mitochondrial morphology rather than mitochondrial dysfunction. The mitochondria of *RECG* and *RECA1* KO plants might change their shape to regulate their activity.

Our results revealed extensive mtDNA rearrangements due to recombination between short repeats in *RECG* KO plants (Figs. 5 and 6). Rearranged mtDNA produced by recombination between relatively long repeats (47–79 bp, most of which exist in introns as direct repeats) accumulated to a high level as detected by DNA gel blot. We found that in such mtDNA rearrangements, for some loci, there was a decrease in normal mtDNA and a significant decrease in intact transcript levels. The decrease in the production of intact transcripts (<10% of WT) was more extensive than the decrease in the levels of normal mtDNA (30%–50% of WT). On the other hand, a substantial amount of chimeric transcript derived from the rearranged mtDNA accumulated in the *RECG* KO mutants. Although the chimeric transcripts were properly spliced, the translation product from the chimeric transcripts had to be largely truncated due to the accidental appearance of stop codon; therefore, it should be defective or dominantly negative to the normal translation products. We also found that aberrant recombination between shorter repeats (<35 bp), which is abundantly scattered in the mitochondrial genome, was induced in both the *RECG* KO and *RECA1* KO mtDNA. This type of recombination occurred at a low frequency, but could produce defective mtDNA with deletion of various loci. Collectively, the *RECG* KO mitochondria are thought to be in a pathological state that includes many kinds of defective mtDNA. These could cause mitochondrial defects via heteroplasmic effect if the normal mtDNA falls below a certain threshold level that is required for normal mitochondria function, as has been conceptually suggested in maize [39]. We found that some kinds of recombined defective mtDNA were highly accumulated in the *recG*-A cells than in *recG*-N cells (Fig. 9), proposing a relationship between the accumulation of the mutated mtDNA and atrophic phenotypes. The cytoplasmic segregation of heteroplasmic *RECG* KO cells with defective mtDNA may have resulted in the biased sorting of the defective mtDNA; when the population of the defective mtDNA exceeds the threshold for the normal mitochondrial function, the cell might exhibit atrophic phenotypes such as those displayed by *recG*-A cells.

A knock-out of the *RECG* gene caused genomic instability due to aberrant recombination among 13–63 bp dispersed repeats in the plastids (Fig. 8) as well as in the mitochondria. Since relatively long repeats ptIR-1 (63 bp) and ptDR-1 (48 bp) exist in the structural genes *rpl16* and *trnG*, and *psaA* and *psaB*, respectively, recombination between the repeats causes truncation or chimerization in these genes (S6A Fig.). In addition, recombination between abundant dispersed short repeats (< 35 bp) causes deletions or inversions in various ptDNA loci. Thus, *RECG* KO plants heteroplasmically contain a substantial proportion of defective ptDNA. On the other hand, most of the plastidic longer repeats (> 40 bp), in which *RECG* KO is assumed to induce recombination more frequently than in shorter repeats, are characteristically located downstream of genes as palindromes (S2 Table), and thus recombinations between them do not disrupt genes directly nor produce defective ptDNA. This property of the plastidic long repeats might alleviate the effect of *RECG* KO on plastid function. The *RECG* KO cells, in particular *recG*-A cells, nevertheless exhibited defects in plastid structure (Fig. 4). Since the amount of the recombined ptDNA was not significantly different between the *recG*-A cells and *recG*-N cells, other ptDNA defect might account for the plastid defects as well as the atrophic phenotypes. Alternatively, the accumulation of starch and oil in the *recG*-A plastids might reflect reduced respiration activity due to mitochondrial dysfunction.

*A*. *thaliana* whirly mutant organelle DNA and *A*. *thaliana MSH1* mutant ptDNA exhibit instability due to recombination between repeats shorter than ∼20 bp [11,14,15], and repeats with similar length are involved in the *RECG* KO organelle DNA instability. This suggests a possibility that RECG, whirly proteins and MSH1 suppress rearrangements in a similar way, however, whirly homologs do not seem to exist in the *P*. *patens* genome [24]. On the other hand, the length of the repeats (8–79 bp) involved in recombination of the mtDNA observed in *RECG* KO mutants differ from those of the *A*. *thaliana MSH1*, *OSB1* and *RECA3* mutants, in which repeats longer than 100 bp are involved [10,12,13]. As *P*. *patens* mtDNA has no repeats longer than 100 bp [6], it is impossible to analyze the effect of *RECG* KO mutants on recombination between repeats longer than 100 bp. Analysis of *RECG* and these genes in *A*. *thaliana* will be needed to elucidate the relationship between *RECG* and these genes.

Some repeated sequences were involved specifically in either *RECG* KO or *RECA1* KO mutant mtDNA rearrangements, while others were involved in both (Fig. 5 and 6). This suggests that *RECG* and *RECA1* suppress recombination in overlapping but also partially distinct ways in mitochondria. We previously proposed a model in which RECA1 prevents aberrant recombination during the process of repairing stalled or collapsed replication forks [6], based on the suggested role of *E*. *coli* RecA [40,41]. Similar to RecA, RecG is thought to function in the processing of stalled replication forks by reversing it in *E*. *coli* [17], which raises the possibility that *P*. *patens* RECG may also be involved in the repair of replication forks stalled by lesions on the template DNA. The lesions might be created by ROS or UV since they can cause replication fork stalling [42,43]. RECG may reverse stalled replication forks perhaps to prevent subsequent fork collapse and DNA double-strand breaks that can induce genomic instability such as aberrant recombination. Furthermore, RECG might also suppress aberrant recombination by stabilizing recombination intermediate between highly homologous sequences, as demonstrated in *E*. *coli* [44]. Differences between the mtDNA rearrangement caused by *RECG* KO and *RECA1* KO might reflect differences in their roles in the repair of impaired replication forks, as studies in *E*. *coli* suggest that RecA and RecG function in replication fork repair in distinct situations [18,45]. On the other hand, similarities in organelle DNA defects of *RECG*, *RECA1*, *MSH1*, whirly, and *OSB1* mutants might suggest that all of these genes are involved in the integrity of replication forks.

In conclusion, our results suggest that RECG suppresses the recombination between the scattered repeats in organelle genomes and that the pathway partly overlaps that of RECA1 in the mitochondria. A future genetic analysis of double KO mutants of these genes and comprehensive analysis of the mtDNA mutations induced in the single and double KO mutants may elucidate the exact relationship between RECA1 and RECG in the maintenance of mitochondrial genome stability. Our results for the *RECG* KO mutants indicate that the mechanisms of the suppression of aberrant recombination are probably common to plastids and mitochondria. Another RECA has been found in plastids of *P*. *patens* [5] and other plant species [25]. The investigation of the functional relationships between RECA and RECG may shed new light on the molecular mechanisms of plastid genome stability.

## Materials and Methods

### Plant materials, growth conditions, and preparation of nucleic acids

*Physcomitrella patens* Bruch & Schimp subsp. *patens* was used in this study. Protonemata of *P*. *patens* were cultured on BCDATG or BCDAT agar medium [46] at 25°C in white light. Growth rate comparisons were performed as described in Odahara *et al*. [6]. Genomic DNA was extracted by the CTAB method [47] from protonemata cultivated for four days after homogenization and inoculation on agar medium. Total RNA was extracted from protonemata using the RNeasy Plant Mini Kit (Qiagen).

### Sequence determination of *RECG* cDNA

RACE was performed according to Hiwatashi *et al*. [48] using total RNA. *RECG* sequence data can be found in the GenBank/EMBL database under accession number AB646798.

### Fluorescent microscopy

*RECG* cDNA corresponding to the full-length 5’UTR and the 284-amino acids of the N-terminal region was amplified by PCR using primers TCCGGATCCTTGCTACACCCTTCTTTCTGCTCCG and CGACCATGGTGCCATCCACAGTGGATTTGTACAGC and fused in frame to the *GFP* gene of p7133-sGFP [49], and the fused gene was expressed under control of E7133 promoter [50]. Similarly, full-length *RECG* coding sequence amplified by primers ACTACATCTAGAGGATCCCCGCGATGGCAATTAGAGGTTGTAG and AAGCTTTCCCATGGCCACCCAGTTTTGTTTATCTAGAGCCTCCAG was fused in frame to N-termini of *GFP* gene and expressed under control of the E7133 promoter. The resulting plasmid was introduced into *P*. *patens* protoplasts, and the protoplasts stained with 125 nM Mito Tracker Orange (Molecular Probes) or 1 μg/ml DAPI were observed with an epifluorescence microscope (AX80, Olympus; Axio Imager 2, Zeiss).

### Complementation assay in *E*. *coli* cells

The *RECG* cDNA, except for a part corresponding to the N-terminal putative signal peptide (374 amino acids), was amplified by PCR using primers CCTCCATGGGAGCTGCTAACTACAAAGATTGTG and CCTCCCGGGCAACATTATCCTAAAGAACCCGGTG, and *E*. *coli recG* was amplified by primers CCCGAATTCACCATGAAAGGTCGGCTGTTAG and CCCCTGCAGTTACGCATTCGAGTAACGTTCCG. Both were placed under the inducible arabinose promoter of pBAD24 [51], and each of the resulting plasmids or pBAD24 was introduced into the *E*. *coli recG* deficient strain HRS2000 [27]. After cultivating the strains to an optical density at 600 nm of 0.5 in L broth medium [52] containing 0.5% arabinose, the cells were appropriately diluted and spread on L broth agar media. Then, they were subjected to UV (254 nm) irradiation and cultivated at 37°C. Colony-forming units (cfu) were counted and the surviving fraction was calculated by dividing the cfu in UV irradiated cells by that in nonirradiated cells.

### Generating the *RECG* gene knockout

To prepare the *RECG* KO construct, fragments of the *RECG* gene were amplified using primers CAGAATGAGAGCTCTGAGTATGGTGTTC and GAGCCGCGGGAGAGGCTAAGCCTGAAACATTGGAG to obtain a 1.6-kb fragment from the 5’ region, and CAGATCGATGCGACAGTAACGCAAGAAGAAGCAC and GAAGGGCCCTGGGGATTAAGTCTTCTAGTTTGCCTG to obtain the 1.6-kb fragment from the 3’ region. Each of them was then introduced into either side of the *nptII* cassette of pTN3 [46]. The resulting plasmid, named pMSD202, was linearized before the transformation. Moss transformation was performed according to Nishiyama *et al*. [46]. The transformants were selected on medium containing G418. To confirm knockout of *RECG* gene, PCR was performed with primers TCCGGATCCTTGCTACACCCTTCTTTCTGCTCCG and GTTGCTCATCCACAACAGCC.

### Transmission electron microscopy

Protonemata cultivated on BCDAT agar medium were fixed with 4% paraformaldehyde and 2% glutaraldehyde in sodium cacodylate buffer, pH 7.4, for overnight at 4°C. Then, cells were fixed with 1% osmium tetroxide in cacodylate buffer, pH 7.4, for 5 h at room temperature. After dehydration in a graded methanol series, the samples were embedded in Epon812 resin. Thin sections were stained with uranyl acetate and lead citrate and observed with JEM-1400 electron microscope (JEOL).

#### DNA gel blot analysis

Total genomic DNA was separated on a 0.7% agarose gel and blotted onto a nylon membrane. To detect nuclear-encoded and mitochondrial-encoded genes, 4 and 0.5 μg of total genomic DNA were used, respectively. Probes were prepared by PCR using the PCR DIG Probe Synthesis Kit (Roche) and the following primers: GCCTAGGAGGGCGCGTTTGGGAAGACG and CCCAGACACATAACTATAGTGCTAGCCG for *nad2*; AACACTTGGGTACATGCCAGCCA and GGACACACGGCTACTATGCGATT for *atp9*; GTGAATGAGTATAAGCTTCGCTGCTCAA and CTATGTATAGCCACTTTGGTAGTGCTTG for *ccmF*; CGGGTTAGGGGTACGACAGATAGCG and TAATACGACTCACTATAGGGCGAGTAGTTCTATCTATCTACCTCTCC for *nad7*; GCGCATGCACATTTCCAAGC and GTAGTTATGCTTCAGATGCTTTGC for *nad7* in S5A Fig.; CTTGAGAAGCGCAACCTGTG and GCTGTGCCTTTGAAGCTTCG for *nad9*; ATCCCTGATCCCAGAATACGACTG and GGCTAAGAGCATGAAGACAGATCC for *nad4*; GTCTGCTGGCAGAAATTCATC and TTGCGCCTTGACCTGGATTC for *rpl2*; AACGAGTCGTACACTAAGC and ATTCGCGGTCGTTCGTATG for *rpl16*; and GGACGAATTTTCCATCTCCAAGG and GGAGGAGTTGCTGTAGATTTACC for *ndhH*. Hybridization of the probes was performed at 37°C, and the membranes were washed in 2x SSC with 0.1% SDS at 25°C and 0.5x SSC with 0.1% SDS at 65°C. Detection of the DIG-labeled probes was performed with Anti-DIG-Alkaline Phosphatase (Roche) and AttoPhos (Promega). All the DNA gel blots were re-hybridized to mitochondrial *rpl2* or plastid *ndhH* probe to estimate organelle DNA copy number (S7 Fig.).

### PCR analysis of organelle DNA

PCR analyses of DNA generated by recombination between repeated sequences were performed using total genomic DNA and the primers listed in S4 Table. Quantitative PCR was performed with the Applied Biosystems 7500 Fast Real-Time PCR System and POWER SYBR Green Master Mix (Applied Biosystems International). qPCR was performed with technical replicates of three independent reactions. Standard PCR analysis was performed in the exponential amplification phase, and the quantity of amplification products was compared using ethidium bromide staining.

### RT-PCR analysis of mitochondrial transcripts

Total RNA extracted from protonemal cells was treated with TURBO DNase (Ambion) to remove residual genomic DNA and then reverse-transcribed using random hexamer. Quantitative RT-PCR was performed using primers listed in S5 Table with the Applied Biosystems 7500 Fast Real-Time PCR System and POWER SYBR Green Master Mix (Applied Biosystems International). Serine threonine protein phosphatase 2a, ST-P 2a, was used as a reference gene [53]. qRT-PCR was performed with technical replicates of three independent reactions. Standard RT-PCR analysis was performed in the exponential amplification phase.

### Assay for evaluation of PCR jumping in qPCR

DNA fragments harboring copy 1 or 2 of ptIR-1 was amplified by standard PCR using primers GGTCCATAAAGGAGCCGTATG and GGGTAGTGGGAATCGAACC for the copy 1 of ptIR-1; CATCCTTCCTCTATGTTGTTTACGA and TCACAAGAAGCCGGATGAAA for the copy 2 of ptIR-1. Relative copy number of the products was analyzed by qPCR using the same primer pairs, and the two fragments were mixed to contain the same copy number of copy 1 or 2 of ptIR-1 as the WT genomic DNA. Then copy number of the DNA recombined between copy 1 and 2 of ptIR-1 were analyzed by qPCR using primers GAATCGAACCCACATCATTAGCT and TATTCACAAGAAGCCGGATGAA and the WT genomic DNA or the mixed DNA fragments as templates.

## Supporting Information

S1 Fig Characteristics of the plant RecG homologs. **A**. Multiple sequence alignment of plant RecG homologs and *E*. *coli* RecG. Protein sequences of *P*. *patens* (AB646798) and *A*. *thaliana* (AT2G01440; AEC05451) RecG homologs, and *E*. *coli* RecG (NP_418109) were aligned by ClustalW2. Identical residues are colored in black. The residues corresponding to candidates for the first and second translation initiation codons are indicated by the arrows. **B**. Localization prediction of plant RecG homologs by TargetP using their N-terminal 130 residues. The scores represent reliability with a maximum score = 1. Pt, plastid; Mt, mitochondrion.
(EPS)

S2 Fig Targeted knockout of the *RECG* gene. **A**. Schematic representation of *RECG* gene targeting. The *RECG* locus, a targeting construct, and the targeted *RECG* locus are depicted. Targeting of the *RECG* gene results in an insertion of the *nptII* cassette into the *RECG* gene so that a large portion of the produced RECG protein is deleted. The boxes and the lines between boxes represent the exons and introns, respectively. *RECG* coding regions are colored in gray. The primers used in (B) are represented by arrows. The *nptII* cassette consists of the cauliflower mosaic virus 35S promoter-driven neomycin phosphotransferase II gene. **B**. PCR analysis of the *RECG* locus. Genomic *RECG* locus was analyzed by PCR with primers showed in (A) and genomic DNA from wild-type (WT) and *RECG* KO lines. The sizes of the DNA bands are indicated on the right of the picture.
(EPS)

S3 Fig Ultrastructural analysis of *RECG* KO protonemal cells. **A**. Light microscopy images of WT and *RECG* KO protonemal cells. *RECG* KO protonemal cells show heterogeneity in growth; atrophic cells (*recG*-A) and relatively normal cells (*recG*-N). **B**. TEM image of an extremely extended mitochondrion observed in a *recG*-6N cell. An arrowhead indicates the extended mitochondrion. **C** and **D**. Serial thin sections (∼80 nm each) of *recG*-6N cell mitochondria observed by TEM. A series of sections of middle part of a mitochondrion (C) and those of edge of another mitochondrion (D). The numbers shown in the right upper of the pictures denote order/total number of the sections. **E**. Inhibition of cell septum formation by an extended plastid in the *recG*-6N cell. **F**. Oil accumulation observed in the *recG*-3A cell. Arrowheads indicate exampes of oil bodies. Bars = 200 μm in (A), 600 nm in (B), 1 μm in (C) and (D), and 5 μm in (E) and (F).
(EPS)

S4 Fig mtDNA rearrangements of the *RECG* KO plants. **A** and **B**. mtDNA configuration at the *atp9* and *ccmF* loci. DNA from wild-type *RECG* KO and *RECA1* KO strains digested with HindIII (A) or NdeI (B) was blotted onto nylon membranes and hybridized with the probes shown below the blots. The expected structures and the length of the major bands are indicated on the right of the blots. **C**. Schematic representation of the repeat structures located at the *atp9* and *ccmF* loci and the flanking region. The repeats (47 bp, 100% identity) are indicated by the white triangles in the boxes. The probes used in (A) and (B), and the HindII or NdeI recognition sites are indicated by thick gray lines and H or N, respectively. **D**. Nucleotide sequences of *ccmF*, *atp9*, and *ccmF-atp9* recombination product. Repeated sequences (47 bp long) are boxed. **E**. Relative copy number of mtDNA loci. The relative copy number of three mitochondrial loci (*rps4*, *nad6* and *rpl2*) per nuclear actin was measured by qPCR. The copy number in the wild-type background was given a value of 1. The data represent mean of three replicates ± SD. All the *RECG* KO and *RECA1* KO values are significantly different from WT values (p<0.05).
(EPS)

S5 Fig Mitochondrial genome and transcript of *RECG* KO plants. **A** to **D**. Quantitative DNA gel blots of mitochondrial *nad7* (A), *nad9* (B), *atp9* (C), and *ccmF* (D) loci. Total genomic DNA digested with EcoRI (A, C and D) or SacII (B) were hybridized with each probes, respectively. The relative intensities of normal mtDNA fragments calculated by ImageJ were shown below the blots. The structures of each loci in A and B are depicted in the right panels. The EcoRI and SacII recognition sites are indicated by E and S, respectively. The positions of the probes used in the blots are indicated by thick gray lines. The boxes represent exons, and the lines between boxes represent introns or noncoding flanking sequences. The 90 bp repeats, which are not included in the length of the segments represented in the scheme, are indicated by black triangles in the boxes. The structures of *atp9* and *ccmF* loci were explained in Fig. 5B. **E**. Sequence chromatogram of *nad7-nad9* chimeric transcript. Reading frame from *nad7* exon 2 encounters termination codon at the beggining of *nad9* exon 2. **F**. qRT-PCR analysis of *atp9* and *ccmF* transcripts. Relative amount of segments of mitochondrial transcripts from exon 2–4 of *atp9* four exons and exon 1–2 of *ccmF* composed of two exons were normalized by that of reference nuclear gene ST-P 2a transcript. WT was given a value of 1. The data represent mean of three replicates ± SD. **G**. Estimation of residual genomic DNA in the qRT-PCR. Relative amount of segment of *nad9* exon 1 was estimated by qPCR using RNA with (+RT) or without (-RT) reverse transcription. The values are <0.0005 in WT (-RT) and *recG*-4 (-RT). N.D., not detected. The data represent mean of three replicates ± SD.
(EPS)

S6 Fig Analysis of plastid DNA in *RECG* KO plants. **A**. Schematic representation of recombination at ptIR-1 and ptDR-1. The boxes represent exons, and the lines between boxes represent introns or noncoding flanking sequences. ptIR-1 and ptDR-1 are indicated by black triangles in the boxes. Primers used in the qPCR assay are represented by arrows. **B**. Relative copy number of ptDNA loci. Relative copy number of three plastidic loci (*rbcL*, *atpA* and *ndhH*) per nuclear actin was measured by qPCR. The copy number in the wild-type background was given a value of 1. The data represent mean of three replicates ± SD. All the *RECG* KO values are significantly different from WT values (p<0.01). **C**. Estimation of ptDNA copy number and plastid number per cell. Relative copy number of three plastidic loci per nuclear actin was measured by qPCR. The number of plastid per second cells was counted by using microscopy. Data are expressed as mean ± SD (n = 3 in qPCR, n = 20 in plastid number). All the *RECG* KO values in ptDNA copy number quantification are significantly different from WT values (p<0.01). **D**. Evaluation of the rate of PCR jumping in the qPCR analysis. Relative copy number of copy1 and copy2 of ptIR-1, and DNA recombined between the copy1 and copy2 were measured by quantitative PCR using primers P1 and P2, P3 and P4, and P5 and P6, respectively. WT genomic DNA or mixture of DNA fragments containing copy1 of ptIR-1 and that containing copy2 was used as templates. The relative copy number in the wild-type genomic DNA was given a value of 1. The data represent mean of three replicates ± SD.
(EPS)

S7 Fig Analysis of organelle DNA loci in the *RECG* and *RECA1* KO mutants. DNA gel blots in the figures were reprobed with mitochondrial *rpl2* probe (A) or plastid *ndhH* probe (B) to estimate the copy number of organelle DNA.
(EPS)

S8 Fig Sequences of organelle recombination products. Junction sequences of the mitochondrial recombination products in Fig. 6D and plastid recombination products in Fig. 8D are represented with their original organelle DNA locus sequences in panel A and panel B, respectively. Numbers in both sides of the sequences represent positions according to the mitochondrial DNA sequence (AB251495) or plastid DNA sequence (AP005672).
(EPS)

S1 Table Mitochondrial short repeats (<35 bp) involved in recombination. List of mitochondrial short repeats (<35 bp) involved in recombination in Fig. 6D.
(DOCX)

S2 Table Repeated sequences (>40 bp) in the *P*. *patens* plastid genome. List of repeated sequences (>40 bp) in *P*. *patens* plastid DNA sequence identified by REPuter.
(DOCX)

S3 Table Plastidic short repeats (<35 bp) involved in recombination. List of plastidic short repeats (<35 bp) involved in recombination in Fig. 8D.
(DOCX)

S4 Table Primers used for PCR analysis. List of primers and their sequences used for PCR analysis.
(DOCX)

S5 Table Primers used for RT-PCR analysis. List of primers and their sequences used for RT-PCR analysis.
(DOCX)
