# Supplementary material for: RECG Maintains Plastid and Mitochondrial Genome Stability by Suppressing Extensive Recombination between Short Dispersed Repeats
Source: PLoS Genet. 2015 Mar 13;11(3):e1005080. doi: 10.1371/journal.pgen.1005080 (PMC4358946; doi:10.1371/journal.pgen.1005080)
Supplement: S1 Table — List of mitochondrial short repeats (<35 bp) involved in recombination in Fig. 6D. (DOCX) [file pgen.1005080.s009.docx]

| **S1 Table. Mitochondrial short repeats (<35 bp) involved in recombination** | | | | | | | |
| --- | --- | --- | --- | --- | --- | --- | --- |
|  |  |  |  |  |  |  |  |
| Reaction  number^1^ | Repeat-1 | |  | Repeat-2 | |  |  |
|  | Length (bp) | Position (bp)^2^ |  | Length | Position | Orientation^3^ | Mismatch (bp) |
| 1 | 21 | 50312 |  | 21 | 60075 | DR | 0 |
| 1 | 8* | 49689 |  | 8* | 60153 | DR | 0 |
| 2 | 18 | 50320 |  | 18 | 65050 | IR | 0 |
| 3 | 15 | 41235 |  | 15 | 57326 | IR | 0 |
| 4 | 13 | 20105 |  | 13 | 74224 | DR | 0 |
| ^1^The PCR reaction numbers corresponding to the images in Figure 6D. | | | | | | | |
| ^2^Smallest number of the position of the repeated sequences corresponding to *P. patens* mtDNA sequence accession number AB251495. | | | | | | | |
|  |  |  |  |  |  |  |  |
| ^3^DR, direct repeat; IR, inverted repeat. | | | | | | | |
| *The repeats from which recombination products were generated unexpectedly. | | | | | | | |
