# Supplementary material for: RECG Maintains Plastid and Mitochondrial Genome Stability by Suppressing Extensive Recombination between Short Dispersed Repeats
Source: PLoS Genet. 2015 Mar 13;11(3):e1005080. doi: 10.1371/journal.pgen.1005080 (PMC4358946; doi:10.1371/journal.pgen.1005080)
Supplement: S2 Table — List of repeated sequences (>40 bp) in P. patens plastid DNA sequence identified by REPuter. (DOCX) [file pgen.1005080.s010.docx]

| **S2 Table. Repeated sequences (>40 bp) in the *P. patens* plastid genome** | | | | | | | |
| --- | --- | --- | --- | --- | --- | --- | --- |
|  |  |  |  |  |  |  |  |
|  | Repeat-1 | |  | Repeat-2 | |  |  |
| No. | Length (bp) | Position (bp)^1^ |  | Length | Position | Orientation^2^ | Mismatch (bp) |
| R1 | 9592 | 85211 |  | 9592 | 113298 | IR | 3 |
| R2 | 78 | 8950 |  | 78 | 8950 | Palindrome | 0 |
| R3 | 77 | 28327 |  | 77 | 28327 | Palindrome | 3 |
| R4 | 64 | 6358 |  | 64 | 6358 | Palindrome | 0 |
| R5 | 64 | 45846 |  | 64 | 45846 | Palindrome | 0 |
| R6 (ptIR-1) | 63 | 61583 |  | 63 | 80847 | IR | 3 |
| R7 | 60 | 40267 |  | 60 | 40267 | Palindrome | 2 |
| R8 | 58 | 4501 |  | 58 | 4501 | Palindrome | 0 |
| R9 | 57 | 18243 |  | 57 | 18243 | Palindrome | 3 |
| R10 | 56 | 65012 |  | 56 | 65012 | Palindrome | 0 |
| R11 | 51 | 16224 |  | 51 | 16224 | Palindrome | 1 |
| R12 (ptDR-1) | 48 | 36675 |  | 48 | 38899 | DR | 3 |
| R13 | 46 | 53115 |  | 46 | 53115 | Palindrome | 0 |
| R14 | 45 | 24335 |  | 45 | 24342 | Palindrome | 3 |
| R15 | 42 | 61924 |  | 42 | 61972 | Palindrome | 2 |
| R16 | 41 | 22472 |  | 41 | 22519 | Palindrome | 3 |
| ^1^Smallest number of the position of the repeated sequences corresponding to *P. patens* ptDNA sequence accession number AP005672. | | | | | | | |
| ^2^IR, inverted repeat; DR, direct repeat. | | | | | | | |
