# Supplementary material for: RECG Maintains Plastid and Mitochondrial Genome Stability by Suppressing Extensive Recombination between Short Dispersed Repeats
Source: PLoS Genet. 2015 Mar 13;11(3):e1005080. doi: 10.1371/journal.pgen.1005080 (PMC4358946; doi:10.1371/journal.pgen.1005080)
Supplement: S3 Table — List of plastidic short repeats (<35 bp) involved in recombination in Fig. 8D. (DOCX) [file pgen.1005080.s011.docx]

| **S3 Table. Plastidic short repeats (<35 bp) involved in recombination** | | | | | | | | |
| --- | --- | --- | --- | --- | --- | --- | --- | --- |
|  |  |  |  |  | |  |  |  |
| Reaction  number^1^ | Repeat-1 | |  | Repeat-2 | | |  |  |
|  | Length (bp) | Position (bp)^2^ |  | Length | Position | | Orientation^3^ | Mismatch (bp) |
| 1 | 34 | 6319 |  | 34 | 80953 | | IR | 3 |
| 1 | 13* | 6342 |  | 13* | 81346 | | IR | 0 |
| 2 | 28 | 41911 |  | 28 | 60192 | | IR | 0 |
| 3 | 19 | 45499 |  | 19 | 81340 | | IR | 0 |
| 3 | 15* | 45896 |  | 15* | 81343 | | IR | 0 |
| 4 | 17 | 28385 |  | 17 | 81465 | | DR | 0 |
| ^1^The PCR reaction numbers corresponding to the images in Figure 8D. | | | | | | | | |
| ^2^Smallest number of the position of the repeated sequences corresponding to *P. patens* cpDNA sequence accession number AP005672. | | | | | | | | |
|  |  |  |  |  |  |  |  |  |
| ^3^DR, direct repeat; IR, inverted repeat. | | | | | | | | |
| *The repeats from which recombination products were generated unexpectedly. | | | | | | | | |
