# Supplementary material for: RECG Maintains Plastid and Mitochondrial Genome Stability by Suppressing Extensive Recombination between Short Dispersed Repeats
Source: PLoS Genet. 2015 Mar 13;11(3):e1005080. doi: 10.1371/journal.pgen.1005080 (PMC4358946; doi:10.1371/journal.pgen.1005080)
Supplement: S4 Table — List of primers and their sequences used for PCR analysis. (DOCX) [file pgen.1005080.s012.docx]

| **S4 Table. Primers used for PCR analysis** | | |
| --- | --- | --- |
|  | | |
| Target |  | Sequence |
| mtIR | forward | TACTAGTCCAATGTCGCCATAAGG |
|  | reverse | ATTCTCGACCACAGGAATTTCC |
| mtR5 | forward | GCCTGCTTAAACATACAGTGG |
|  | reverse | GTCAAAAGTTACTTCATAGAGCCG |
| mtR11 | forward | AGGACTATGTGCCAAACAGAC |
|  | reverse | AGAGTCAAGAATTCCATCGCAG |
| mtR12 | forward | CCTGAGTAAGCCCATTTCCG |
|  | reverse | GCCAGAACACAAAGGTGACTAG |
| mtR13 | forward | GCCTGCTTAAACATACAGTGG |
|  | reverse | CACACTGGTTTGTACACGAATG |
| mtR18 | forward | TGACACGGGAAATCTGACTTC |
|  | reverse | AAAATTTAATTGATGCCAGCTATGC |
| mtR19 | forward | ACTGAAGTGGATTTCACCGG |
|  | reverse | GAACACTGTTATGCTTTCGGC |
| mtR22 | forward | TTAAATAGAGTGGAACTTTCTCAGA |
|  | reverse | ACGAGCCGCAGTCAATG |
| *nad7-nad9* rec | forward | CATAACTACACACTCACACGATCT |
|  | reverse | TGCCAGCGCTTTCCTTT |
| *ccmF-atp9* rec | forward | TTCGGTTTTCAGGTTCCCC |
|  | reverse | GCGCACACTAAATACGTTTCC |
| ptIR-1 | forward | GAATCGAACCCACATCATTAGCT |
|  | reverse | TATTCACAAGAAGCCGGATGAA |
| ptDR-1 | forward | CGTGGAGGATTAAATCCAGTTAC |
|  | reverse | TGCCCACGGCCTAAACGA |
| *rps4* | forward | TTCAGAGTAACCGAATATGGAGAATAAA |
|  | reverse | GAGGTTCATATAATACCACGGCTTTT |
| *nad6* | forward | CGTGCTAAAAATCCAGTCCATTC |
|  | reverse | AGCAAAGAAGTCAAGACCTAACAAAAC |
| *rpl2* | forward | TAGGCAGACGTCCCATTGTTC |
|  | reverse | TCCTTCGCCTCCTCCATGAG |
| *rbcL* | forward | TTCCTTCGCGAGCAAGGT |
|  | reverse | GCTAACAGGGTTGCTTTAGAAGCT |
| *atpA* | forward | CAGATGGCTTGATGAGAGGAATG |
|  | reverse | GAGTAGCTTCACCAACTGGAACAC |
| *ndhH* | forward | AGTAATCCCATCGCGTGACAT |
|  | reverse | AGGTATGGAAAAAATCGCTGAAAA |
| actin | forward | CAACCGTCTTCTGTGTCTAGGTC |
| (S4E and S6B Figs.) | reverse | GAAACCGGCCTGCATTACATG |
| Fig. 6D-1 | forward | ATCCCTGATCCCAGAATACGACTG |
|  | reverse | AGCAAAGAAGTCAAGACCTAACAAAAC |
| Fig. 6D-2 | forward | GGCTAAGAGCATGAAGACAGATCC |
|  | reverse | CCATAAAGCGCGAACCAACATCCA |
| Fig. 6D-3 | forward | ATTCTCGACCACAGGAATTTCC |
|  | reverse | ATGGCTTGCAGTCCGCTAGAACAA |
| Fig. 6D-4 | forward | CTATGTATAGCCACTTTGGTAGTGCTTG |
|  | reverse | CGGGCTGGTCCAGTAATTCTAA |
| *rpl2* | forward | GTCCAGGAAAGCCCAGAGAAG |
| (Fig. 6D) | reverse | CCTAAGGGACCTTTGGCCTTC |
| Fig. 8D-1 | forward | GCCTTCTATGATTGGTGAACCAGCA |
|  | reverse | GCTTAGTGTACGACTCGTTAAAATCAATG |
| Fig. 8D-2 | forward | GGGGGTACCCAAATCCCGTCTCCGCTAAAGC |
|  | reverse | GGGGGTACCTGATCCTGGTCGTAATCCTGGACG |
| Fig. 8D-3 | forward | CCGTGGATATGGCCGAGATTCAAT |
|  | reverse | GCTTAGTGTACGACTCGTTAAAATCAATG |
| Fig. 8D-4 | forward | CAGATGGCTTGATGAGAGGAATG |
|  | reverse | GCTTAGTGTACGACTCGTTAAAATCAATG |
| *ndhH* | forward | GGACGAATTTTCCATCTCCAAGG |
| (Fig. 8D) | reverse | GGAGGAGTTGCTGTAGATTTACC |
| actin | forward | CATGTTCGAGACGTTCAACGTGCCG |
| (Fig. 6D&8D) | reverse | GATGGACCAGATTCATCGTACTCGC |
