# Supplementary material for: RECG Maintains Plastid and Mitochondrial Genome Stability by Suppressing Extensive Recombination between Short Dispersed Repeats
Source: PLoS Genet. 2015 Mar 13;11(3):e1005080. doi: 10.1371/journal.pgen.1005080 (PMC4358946; doi:10.1371/journal.pgen.1005080)
Supplement: S5 Table — List of primers and their sequences used for RT-PCR analysis. (DOCX) [file pgen.1005080.s013.docx]

| **S5 Table. Primers used for RT-PCR analysis** | | |
| --- | --- | --- |
|  | | |
| Target |  | Sequence |
| *nad7* exon 1 | forward | CTCAACATCCTGCTGCTCAT |
|  | reverse | GTTCCGCGCGTTCTACA |
| *nad7* exon 1-2 | forward | CTCAACATCCTGCTGCTCAT |
|  | reverse | TTCTGTGCCTCTATGAAGTAATCC |
| *nad7* exon 1-3 | forward | ACCGCATATTGGATTACTTCATAGA |
|  | reverse | TCTTGGGCCATCATAGAAACATAA |
| *nad7* exon 2-3 | forward | CAAGCTTTACCTTATTTTGATCGTTTAG |
|  | reverse | GCTCGTAATGGTACCTCACAA |
| *nad7* exon 3 | forward | CAGGCGTATGCTGGGATTT |
|  | reverse | CGGTCATAACAATCTCCTCTGG |
| *nad9* exon 1 | forward | ATACCAATCCTGATTACCTATTTCAA |
|  | reverse | GAGAAGGATAATCAACTCCACAAA |
| *nad9* exon 1-2 | forward | TGTGGAGTTGATTATCCTTCTCG |
|  | reverse | CTGCCGAACAAATTGGAGTTATT |
| *nad9* exon2 | forward | AGTCAGTATATTTCCGTCAGCC |
|  | reverse | GATGACCCTCAAAACCATAATCTG |
| *atp9* exon 2-4 | forward | TGGAGCAGGAGCAGCTA |
|  | reverse | GGATTTCGCGCAACAGAATG |
| *ccmF* exon 1-2 | forward | ACATGGAGGAGTTTGCATCTT |
|  | reverse | CTCGCAAACAACAATGCTCTC |
| *nad5* | forward | TTGCATACTCGGTTACCTGATG |
|  | reverse | GGGAGCACCTTGCTATCATAAA |
| *nad6* | forward | CGTGCTAAAAATCCAGTCCATTC |
|  | reverse | AGCAAAGAAGTCAAGACCTAACAAAAC |
| *rps4* | forward | TTCAGAGTAACCGAATATGGAGAATAAA |
|  | reverse | GAGGTTCATATAATACCACGGCTTTT |
| *rpl2* | forward | TAGGCAGACGTCCCATTGTTC |
|  | reverse | TCCTTCGCCTCCTCCATGAG |
| ST-P 2a | forward | GTCTAGTTAGTCCTTTGGTCCT |
|  | reverse | GCCTATTTCTATAATGACTCCGT |
| *nad7-nad9* chimera | forward | ACCGCATATTGGATTACTTCATAGA |
|  | reverse | GATGACCCTCAAAACCATAATCTG |
| actin | forward | CATGTTCGAGACGTTCAACGTGCCG |
|  | reverse | GATGGACCAGATTCATCGTACTCGC |

**References**

1. Terasawa K, Odahara M, Kabeya Y, Kikugawa T, Sekine Y, et al. (2007) The mitochondrial genome of the moss Physcomitrella patens sheds new light on mitochondrial evolution in land plants. Mol Biol Evol 24: 699-709.

2. Sugiura C, Kobayashi Y, Aoki S, Sugita C, Sugita M (2003) Complete chloroplast DNA sequence of the moss Physcomitrella patens: evidence for the loss and relocation of rpoA from the chloroplast to the nucleus. Nucleic Acids Res 31: 5324-5331.
